# Between Accessibility and Reliability: High Confidence, Low Control in General-Purpose Multimodal Models for Hip Fracture Radiograph Interpretation

**DOI:** 10.3390/jcm15134919

**Published:** 2026-06-24

**Authors:** Hadar Gan-Or, Shaked Ankol, Guy Ben Arie, Itay Ashkenazi, Yaniv Warschawski

**Affiliations:** 1 Department of Orthopedic Surgery, Hillel Yaffe Medical Center, Hadera 3820302, Israel; 2 Ruth and Bruce Rappaport Faculty of Medicine, Technion—Israel Institute of Technology, Haifa 3525433, Israel; 3 Department of Orthopedic Surgery, Tel Aviv Sourasky Medical Center, Tel Aviv 6423906, Israel; guybenarie@gmail.com (G.B.A.);; 4 Gray Faculty of Medical and Health Sciences, Tel Aviv University, Tel Aviv 6997801, Israel

**Keywords:** hip fracture, pelvic radiography, AI-assisted radiograph interpretation, fracture classification, multimodal large language models, prompt sensitivity, model confidence, AI safety

## Abstract

**Background**: Dedicated artificial intelligence (AI) systems for fracture detection already exist, yet general-purpose multimodal models are increasingly accessible to clinicians despite not being developed or formally validated as medical devices. Their behavior in focused orthopedic imaging tasks remains insufficiently characterized. **Purpose**: To characterize how two accessible general-purpose multimodal models interpret AP pelvis radiographs with hip fractures, focusing on context dependence, overconfidence, and complementary error patterns within a surgically confirmed positive-only cohort. This was a behavioral characterization study of a fracture-positive cohort, not a diagnostic accuracy evaluation. **Methods**: In April 2026, we retrospectively studied 214 surgically confirmed hip fractures on AP pelvis radiographs using two general-purpose multimodal models under six prompting conditions. In runs A–D, the models were explicitly told that a hip fracture was present and were asked to classify it; in runs E–F, they were not told whether a hip fracture was present. Each image was rerun de novo in a separate chat session through vendor APIs using a fixed base prompt and no image preprocessing. We recorded hip-fracture detection, correct laterality, coarse fracture pattern, intracapsular displacement, AO/OTA grading, subtrochanteric identification, and self-reported confidence. Because the cohort contained hip fractures only, we report fracture-detection rates and classification performance within a positive-only cohort rather than full diagnostic-accuracy metrics. **Results**: Using the more conservative endpoint of hip-fracture detection with correct laterality, GPT-5.4 was correct in 79.0% and 86.4% of cases in runs E and F, whereas Gemini was correct in 80.4% and 93.5%, respectively. When outputs from both models were combined, this endpoint reached 89.7% in run E and 96.7% in run F, indicating complementary rather than redundant error patterns. Incorrect laterality cues markedly degraded performance, from 90.7% to 66.4% in GPT-5.4 and from 97.7% to 57.0% in Gemini. Performance remained limited for treatment-relevant subtyping, particularly AO/OTA grading and subtrochanteric identification. Both models frequently remained highly confident when wrong, and self-reported confidence did not reliably distinguish correct from incorrect outputs. **Conclusions**: Accessible general-purpose multimodal models showed partial capability for coarse hip-fracture interpretation, but they remained context-sensitive, unreliable for treatment-relevant subtyping, and highly confident even when incorrect. Their complementary error patterns are hypothesis-generating rather than evidence of clinical readiness. On the basis of these findings, we do not support unvalidated or uncontrolled clinical use of such models. As access to these tools expands, explicit usage boundaries, minimum performance expectations, repeated local revalidation, and sustained human oversight become increasingly necessary.

## 1. Introduction

Artificial intelligence (AI) applications for fracture detection on radiographs are no longer hypothetical. Systematic reviews suggest that AI can achieve diagnostic performance comparable to clinicians in fracture detection overall, and that commercially available fracture-detection products show good diagnostic accuracy across multiple anatomical regions, particularly when used as an adjunct to human interpretation [1,2]. Within hip-fracture care specifically, a dedicated meta-analysis found that AI models for hip radiographs have shown promising performance and, in selected studies, error rates comparable to expert readers [3].

Hip fracture remains a clinically attractive target for AI because missed or delayed diagnosis can alter treatment timing, increase morbidity, and affect downstream outcomes. Consequently, dedicated hip-fracture algorithms have already been developed for binary detection and subclassification, including deep-learning systems trained specifically to identify hip fracture and functional subtypes from radiographs [4], models designed for difficult entities such as nondisplaced femoral neck fractures [5], and reader-assistance studies showing improved performance with commercial AI support in pelvic and hip trauma workflows [6,7,8]. At the systems level, NICE now recommends selected fracture-detection AI products for evidence-generating use in urgent care, explicitly positioning them as adjuncts rather than replacements for standard clinical care [9].

At the same time, a different and largely unregulated reality has emerged: general-purpose multimodal models are becoming widely accessible to clinicians, researchers, and even lay users. Unlike dedicated fracture-detection products, these models are not built around a narrow orthopedic task, are not necessarily developed under medical-device frameworks, and can be prompted flexibly with text, images, or combinations of both. This changes the practical question. The key issue is no longer whether AI can detect fractures in principle, but how accessible general-purpose models behave when applied to a real orthopedic imaging task, how much their output depends on the prompt and the clinical story, and whether different models make overlapping or complementary errors. Our aim was not to validate such tools for clinical use, but to obtain a structured view of how they behave in a focused orthopedic imaging task.

These questions also raise a broader implementation and governance problem. Early clinical evaluation of AI decision-support systems is now expected to be reported transparently [10], and reporting standards for AI interventions are increasingly formalized [11]. The World Health Organization emphasizes accountability, transparency, and protection from harm in health AI [12], while Royal College of Radiologists guidance stresses local evaluation, minimum deployment standards, and post-deployment monitoring for imaging AI [13,14]. Regulators have likewise introduced principles for change control and transparency in AI-enabled medical devices [15,16]. Yet these guardrails were developed largely for dedicated medical-device pathways. General-purpose multimodal models can already be used clinically without equivalent local validation, version control, or workflow safeguards.

The present study was designed as a mapping study rather than a clinical validation trial. Using a fracture-only cohort of surgically confirmed hip fractures, we evaluated two accessible general-purpose multimodal models under six prompting conditions. A positive-only cohort was considered sufficient for this concept-driven objective because the aim was to characterize behavior within confirmed fracture cases, not to estimate full diagnostic accuracy in an unselected population. The study was not designed as a head-to-head comparison against clinicians or dedicated fracture-AI products, but as a characterization study of model behavior, context dependence, complementary error patterns, and the models’ ability or failure to express uncertainty when interpretation became difficult.

## 2. Methods

Study design and case selection. We performed a retrospective concept-focused characterization study using AP pelvis radiographs from patients with surgically confirmed hip fractures. The source pool initially comprised 241 cases; 27 were excluded because of prior pelvic or hip surgery, pathologic fracture, poor image quality, or technically inadequate radiographs, leaving an analytic cohort of 214 cases. Each included radiograph necessarily contained sufficient proximal femur and hip anatomy for hip-fracture characterization and exclusion of major alternative proximal femoral fracture patterns, as verified against operative findings. Image collection and model evaluation were performed in April 2026 through vendor APIs. Ground truth was derived from operative records. Hip-fracture classification, including assessment of intracapsular displacement, was performed independently by 2 senior orthopedic surgeons; disagreements were resolved by consensus discussion with a third senior orthopedic surgeon, yielding a single reference standard per case. Because the final labels reflect a consensus process rather than fully independent ratings, inter-observer agreement (e.g., kappa) was not computed. The final analytic cohort included 214 cases (mean age, 82 years; 126 women [58.9%] and 88 men [41.1%]; 107 right-sided and 107 left-sided hip fractures). Fracture distribution was internally consistent: 81 hip fractures were intracapsular and 133 were extracapsular, including 100 intertrochanteric and 33 subtrochanteric fractures. Among intracapsular hip fractures, 70 were displaced and 11 nondisplaced. No image preprocessing was performed (Table 1).

Models and prompting strategy. Two general-purpose multimodal models were evaluated through vendor APIs: OpenAI gpt-5.4 and Google gemini-3.1-pro. Each case was processed 6 times by each model in April 2026, and every run was performed de novo in a completely new chat session to minimize carryover from prior responses. A fixed base prompt was used, with only the prespecified run-specific context varying. In runs A-D, the models were explicitly told that a hip fracture was present and were asked to classify its characteristics. In runs E-F, the models were not told whether a hip fracture was present and therefore had to decide whether a hip fracture existed before classifying it. Runs B, C, and F supplied correct laterality; run D deliberately supplied incorrect laterality as a safety probe. All model outputs were stored verbatim in structured JSON format [17] (Table 2).

Outcomes. We recorded hip-fracture detection (runs E–F only), fracture side, coarse fracture pattern (intracapsular versus extracapsular), intracapsular displacement (displaced versus nondisplaced), AO/OTA grade for intertrochanteric fractures, extracapsular location (intertrochanteric versus subtrochanteric), and self-reported confidence. Model outputs were evaluated at three hierarchical levels: (1) detection—whether the model identified a hip fracture; (2) laterality—whether it correctly identified the affected side; and (3) pattern classification—whether it correctly categorized the fracture type, including coarse pattern, displacement, and AO/OTA subclassification. For runs E and F, correct laterality over all 214 cases was treated as the more conservative combined endpoint because it required both recognition of a hip fracture and correct side assignment.

Scoring rules. Laterality and coarse fracture pattern were evaluated across all 214 hip-fracture cases. For intracapsular hip fractures, the treatment-relevant subtyping task was a simple dichotomous classification of displaced versus nondisplaced fracture. For extracapsular hip fractures, the treatment-relevant subtyping tasks were AO/OTA grading within the intertrochanteric subset and identification of subtrochanteric location within the extracapsular subset. Any missing, uncertain, or not-applicable answer was counted as incorrect for the relevant endpoint. In runs E and F, if a model failed to identify a hip fracture, all downstream fields were treated as incorrect. Union analyses were descriptive and were used to illustrate complementary error patterns rather than to define a deployable combined system.

Statistical analysis. Statistical analysis was performed in R (version 4.5.3; R Foundation for Statistical Computing, Vienna, Austria). For all major proportions, we calculated Wilson 95% confidence intervals. Pre-specified paired comparisons were assessed using McNemar tests, with Holm correction applied across the family of planned comparisons. Inter-model agreement was summarized with Cohen’s kappa, and weighted kappa was used for AO/OTA because it is an ordered 3-level variable. Confidence behavior was examined with Mann–Whitney U testing and descriptive confidence-bin summaries. Because the cohort contained hip fractures only, we did not calculate specificity, predictive values, or full diagnostic-accuracy metrics; instead, we report hip-fracture detection rates and classification performance within a positive-only cohort. To assess output repeatability, a convenience subset of 25 cases was re-evaluated five times each by both models under the Run E (image-only) condition, retaining all original API parameters (see Appendix A for supplemental methodological notes; Appendix B for details). Within-prompt agreement across the five repeats was quantified with Fleiss’ kappa and Krippendorff’s alpha for categorical classification fields and with the intraclass correlation coefficient (ICC, two-way agreement, single measures) for self-reported confidence scores. Full consistency was defined as identical output across all five repeats. Between-model consistency rates were compared with Fisher’s exact test.

## 3. Results

Overall, the two models showed measurable ability for coarse hip-fracture interpretation, but their strengths differed and their limitations remained substantial. The most clinically relevant endpoint in the independent detection runs was the conservative combined measure of hip-fracture detection with correct laterality. Results are presented beginning with the independent detection conditions (runs E and F), which represent the more clinically demanding scenario, followed by the classification-only conditions (runs A–D) in which the models were told that a fracture was present.

Gemini showed higher hip-fracture detection rates than GPT-5.4 in both independent detection runs, and this difference was statistically supported. Using the more conservative endpoint of hip-fracture detection with correct laterality, union performance reached 89.7% in run E and 96.7% in run F. Detection-only union values are shown in Table 3 for completeness and should be interpreted descriptively rather than as evidence of a deployable combined system.

Clinical context had a mixed effect. Correct context improved laterality in both models, but the most striking result was the wrong-side experiment. Laterality accuracy dropped dramatically in run D in both models, with larger degradation in Gemini than in GPT-5.4. These paired effects were highly significant and indicate that both models can be pulled away from the image by incorrect contextual information (Table 4).

The wrong-side experiment revealed absolute drops of 24.3 percentage points for GPT-5.4 and 40.7 percentage points for Gemini, with 52 and 87 cases, respectively, switching from correct to incorrect laterality assignment when the laterality cue was intentionally wrong. Run D is therefore best understood as a safety stress-test rather than as just another performance condition.

Performance in treatment-relevant subtyping remained limited. GPT-5.4 generally outperformed Gemini in intracapsular displacement, and only GPT-5.4 showed a significant displacement improvement with full context. In contrast, Gemini was numerically superior for subtrochanteric identification and AO/OTA classification, but these tasks remained clinically unreliable overall. Agreement statistics supported this impression: inter-model kappa was only fair for coarse pattern classification and near zero for displacement and AO/OTA, indicating that disagreement was common in the very tasks where precision would matter clinically.

Table 5 is intentionally limited to the statistical results that directly support the core interpretation of the study: context can help broad tasks, wrong context can sharply mislead both models, Gemini outperformed GPT-5.4 in independent hip-fracture detection, and only GPT-5.4 showed a significant context-related gain for intracapsular displacement.

In the repeatability sub-study (25 cases, five repeats per model, Run E), GPT-5.4 demonstrated high within-prompt agreement: Fleiss’ κ was 0.942 (95% CI 0.818–1.000) for fracture detection, 0.949 (0.859–1.000) for laterality, and 0.879 (0.791–0.967) for fracture pattern (all *p* < 0.001). Krippendorff’s α yielded closely concordant values (0.943, 0.949, 0.879). Full consistency across all five repeats was observed in 21 of 25 cases (84%). Self-reported confidence scores were stable across repeats (ICC = 0.919, 95% CI 0.863–0.959). Gemini 3.1 Pro showed substantially lower repeatability: Fleiss’ κ was 0.461 (0.337–0.585) for detection, 0.668 (0.565–0.772) for laterality, and 0.415 (0.312–0.518) for pattern (Krippendorff’s α: 0.462, 0.669, 0.416). Full consistency was observed in only 10 of 25 cases (40%). Although Gemini reported higher mean confidence (96.9 vs. 89.8, *p* < 0.001 by Mann–Whitney U), its confidence scores showed near-zero reliability across repeats (ICC = 0.147, 95% CI 0.005–0.359). The difference in full-consistency rates between models was significant (Fisher’s exact test, *p* = 0.003).

## 4. Discussion

Because the cohort included only surgically confirmed fractures, the findings describe model behavior within positive cases and should not be interpreted as measures of diagnostic accuracy in a mixed population. Within a surgically confirmed fracture-only cohort, two accessible general-purpose multimodal models showed real but bounded capability for coarse hip-fracture interpretation. The main contributions of the study are therefore not that these models are clinically ready, nor that one has ‘won’ a benchmark, but that they reveal a characteristic profile: partial capability in broad tasks, substantial context dependence, major vulnerability to wrong contextual cues, confidence that did not reliably match correctness, and complementary error patterns that could inform future safety-net research. All results reported here are tied to specific, version-locked model snapshots (GPT-5.4, resolved to gpt-5.4-2026-03-05; Gemini 3.1 Pro Preview) evaluated in April 2026; subsequent model updates may yield different performance profiles.

The first important finding is that general-purpose models can detect a large proportion of hip fractures even without an explicit fracture cue. This is noteworthy because the fracture-detection literature has largely focused on dedicated tools trained for narrow tasks [3,4,5,6,7]. Our models, by contrast, were not designed as hip-fracture devices, yet still achieved meaningful fracture-detection rates within a positive-only cohort. However, this should not be confused with clinical readiness. Dedicated systems are typically studied as adjunctive tools, often with explicit regulatory, workflow, and implementation framing [2,6,7,9,18,19]. The present work instead concerns accessible general-purpose models that can be invoked ad hoc, outside the usual guardrails of device-specific validation.

The second major finding is that output depended strongly on context. Correct context improved laterality in both models, but incorrect laterality dramatically degraded performance, with no compensatory evidence that either model ‘resisted’ the misleading hint. This result is central because it shows that these systems are not simply extracting visual features from the radiograph; rather, they are integrating image information with prompt framing and clinical narrative. In clinical reality this is both the appeal and the hazard of multimodal systems. A model that benefits from context may also inherit clerical errors, anchoring effects, or flawed histories. That problem is much less visible in traditional image-only fracture-AI studies and deserves explicit treatment in future evaluations of multimodal models [8,10,11]. However, the models’ behavior in run D could also be interpreted as rational weighting of an authoritative-sounding clinical prompt rather than a simple failure to “resist” misleading input; the safety concern remains, but the mechanism may reflect appropriate context integration applied to incorrect information.

A third key observation is the gap between coarse interpretation and treatment-relevant subtyping. In our data, both models were distinctly weaker for displacement, AO/OTA grading, and subtrochanteric identification than for broad tasks such as laterality or coarse pattern classification. That distinction matters clinically. A model that can say ‘fracture likely present’ is not equivalent to a model that can support operative planning. Dedicated studies have already shown that narrow, task-specific algorithms can reach stronger performance on hip-fracture classification problems than either general-purpose model studied here [4,5,6,7]. For example, Marullo et al. [20] achieved approximately 90% accuracy for AO/OTA 31A/B classification using a dedicated YOLOv8 architecture, substantially exceeding the 15–46% subclassification rates observed in Runs E–F with the general-purpose models in the present study. Notably, even among orthopedic surgeons, the inter-observer reliability of AO/OTA subclassification is only fair (kappa 0.38–0.48) [21], suggesting that fine-grained fracture typing is inherently challenging regardless of whether it is performed by humans or AI. The most defensible interpretation, therefore, is not that general-purpose models rival dedicated orthopedic AI, but that they exhibit a ceiling that becomes obvious once morphologically and surgically meaningful subclassification is required.

The fourth major finding is that the models were frequently confident when wrong. This point deserves particular emphasis. The problem is not simply that confidence was high in absolute terms, but that the displayed confidence often remained high even for incorrect answers and therefore could not be relied on as a practical safeguard. That pattern is especially concerning for less experienced users, who may mistake confident phrasing for clinical reliability. In other words, the hazard is not only error, but error delivered with persuasive certainty.

The complementary-error findings are intriguing but should be interpreted conservatively. In our fracture-only cohort, the more conservative combined endpoint of hip-fracture detection with correct laterality reached 96.7% in run F, and kappa in the detection runs remained low, indicating that the two models often failed on different cases. This is a strong signal for future research on safety-net or second-reader workflows. Nevertheless, a union analysis is not a clinical system. Because our cohort contained no non-fracture radiographs, we could not estimate false-positive burden or net clinical benefit. A combined rule that improves hip-fracture detection might still create an unacceptable number of false alerts in real-world practice. The current finding therefore supports further study of model complementarity; it does not justify deployment. More broadly, all performance values reported in this study—including union metrics approaching 96–99%—describe exploratory observations within a fracture-positive cohort and should not be equated with clinically deployable diagnostic accuracy.

An exploratory repeatability analysis (25 cases, five repeats, Run E) showed that GPT-5.4 produced highly consistent outputs across repeats (Fleiss’ κ ≥ 0.88), whereas Gemini 3.1 Pro showed only moderate agreement (κ 0.42–0.67). This difference may partly reflect the different API sampling settings used for each model rather than intrinsic model differences alone. Nonetheless, the finding suggests that single-run accuracy may not fully characterize LLM reliability, and future evaluations should consider reporting repeatability alongside accuracy.

Finally, our results highlight a broader governance problem. Dedicated medical-device pathways increasingly recognize the need for transparent reporting, predefined change management, minimum deployment standards, and post-deployment monitoring [10,11,12,13,14,15,16]. General-purpose commercial models do not fit neatly into that framework. Their training data, internal logic, and update processes are only partially visible to end users, and a performance study at one time point cannot guarantee that the same behavior will persist after future version updates. Defining acceptable conditions for clinical use of such tools remains an open governance challenge that warrants dedicated investigation.

These findings should be read alongside several limitations. The most important is the positive-only design, which prevented estimation of false-positive rates, predictive values, or true screening utility. That limitation is acceptable for the concept-focused aim of the present study, but it necessarily distinguishes this work from a mixed positive–negative diagnostic-accuracy cohort. In particular, the absence of fracture-negative cases introduces spectrum bias: the models were never required to distinguish fractures from normal anatomy or mimicking pathology, so the reported detection rates cannot be extrapolated to clinical populations with realistic fracture prevalence. Future studies incorporating negative controls will be needed to estimate specificity, predictive values, and screening utility. We also studied AP pelvis radiographs that included sufficient proximal femur and hip anatomy for fracture characterization, without lateral views or advanced imaging. Lateral hip radiographs are seldom obtained in the acute emergency setting because of patient pain and limited additional diagnostic yield [22]; the use of AP radiographs alone therefore reflects routine clinical practice rather than a methodological shortcoming. Furthermore, because the cohort comprised only surgically confirmed fractures, nondisplaced and occult fractures—such as femoral neck stress fractures, which are frequently missed on plain AP radiographs and require MRI for reliable diagnosis [23]—were not represented, and the models’ ability to detect such subtle pathology remains unknown. We did not compare model performance directly with radiologists, orthopedic trainees, or commercial dedicated fracture-AI systems on the same cases. Direct head-to-head comparison with human readers on the same image set would strengthen future work but was beyond the scope of this behavioral characterization study, which focused on model behavior rather than diagnostic superiority. In addition, the study examined two model versions at a single point in time; performance may differ with subsequent updates. Ground-truth labels were derived by consensus among senior surgeons rather than by fully independent classification, so inter-observer kappa could not be reported. Although several comparisons yielded very small *p*-values, the absolute performance differences were sometimes modest; statistical significance in this sample should not be conflated with clinical meaningfulness. Taken together, these limitations argue for caution, not dismissal. Its value lies precisely in showing what becomes visible when accessible general-purpose multimodal models are evaluated not as polished medical devices, but as tools that clinicians can already reach for.

## 5. Conclusions

Accessible general-purpose multimodal models showed partial capability for coarse hip-fracture interpretation, but their outputs were strongly shaped by clinical context, vulnerable to misleading information, limited in treatment-relevant subtyping, and often highly confident even when wrong.

More broadly, these models are commercially updated black boxes rather than validated orthopedic devices. In the absence of explicit usage rules, ongoing local validation, and well-defined human oversight, even apparently strong broad-pattern performance should not be mistaken for clinical legitimacy. Because internal model behavior and future version changes are not fully transparent to end users, performance at one time point cannot be assumed to remain stable after later updates. Without such safeguards, unvalidated clinical use of these tools may pose patient safety risks; establishing formal governance conditions therefore remains an urgent priority.

## Figures and Tables

**Table 1:** Baseline characteristics of the analytic fracture cohort.

Characteristic	*n*/Value	%
Total cases	214	
Age, mean (years)	82	
Women	126	58.9
Men	88	41.1
Right-sided fractures	107	50.0
Left-sided fractures	107	50.0
Extracapsular	133	62.1
Intertrochanteric	100	46.7
31-A1 (simple)	34	34.0 of intertrochanteric
31-A2 (multifragmentary)	37	37.0 of intertrochanteric
31-A3 (reverse oblique)	29	29.0 of intertrochanteric
Subtrochanteric	33	15.4
Intracapsular	81	37.9
Displaced	70	86.4 of intracapsular
Nondisplaced	11	13.6 of intracapsular

All fractures were surgically confirmed. AO/OTA percentages are of intertrochanteric fractures (*n* = 100). Intracapsular displacement percentages are of intracapsular fractures (*n* = 81).

**Table 2:** Prompting conditions and intended purpose.

Run	Fracture Cue	Laterality Hint	Full Clinical Context	Purpose
A	Yes	No	No	Image-only baseline classification
B	Yes	Correct	No	Effect of correct laterality hint
C	Yes	Correct	Yes	Effect of richer clinical context
D	Yes	Incorrect	No	Safety test: susceptibility to wrong context
E	No	No	No	Independent fracture detection and classification
F	No	Correct	No	Detection with minimal correct context

In runs A–D, models were explicitly told that a hip fracture was present and were asked to classify it. Runs E–F instead required the model first to decide whether a hip fracture was present. Run D was deliberately designed as a wrong-context stress test.

**Table 3:** Primary performance in the independent hip-fracture detection conditions (runs E and F).

Metric	GPT E	Gemini E	Union E	GPT F	Gemini F	Union F
Detection (/214)	188/214	202/214	207/214	190/214	208/214	212/214
	87.9%	94.4%	96.7%	88.8%	97.2%	99.1%
	(82.8–91.6)	(90.5–96.8)	(93.4–98.4)	(83.9–92.3)	(94.0–98.7)	(96.7–99.7)
Detection + correct laterality (/214)	169/214	172/214	192/214	185/214	200/214	207/214
	79.0%	80.4%	89.7%	86.4%	93.5%	96.7%
	(73.0–83.9)	(74.5–85.1)	(84.9–93.1)	(81.2–90.4)	(89.3–96.1)	(93.4–98.4)
Pattern (/214)	152/214	157/214	187/214	156/214	160/214	189/214
	71.0%	73.4%	87.4%	72.9%	74.8%	88.3%
	(64.6–76.7)	(67.1–78.8)	(82.3–91.2)	(66.6–78.4)	(68.5–80.1)	(83.3–92.0)
Displacement (/81)	31/81	27/81	-	29/81	31/81	-
	38.3%	33.3%		35.8%	38.3%	
	(28.4–49.2)	(24.0–44.1)		(26.2–46.7)	(28.4–49.2)	
AO/OTA (/100)	23/100	40/100	-	23/100	37/100	-
	23.0%	40.0%		23.0%	37.0%	
	(15.8–32.2)	(30.9–49.8)		(15.8–32.2)	(28.2–46.8)	
Subtrochanteric (/33)	7/33	15/33	-	5/33	12/33	-
	21.2%	45.5%		15.2%	36.4%	
	(10.7–37.8)	(29.8–62.0)		(6.7–30.9)	(22.2–53.4)	

**Table 4:** Effect of context and wrong laterality on selected classification outcomes.

Metric	Model	Run A	Run B	Run C	Run D	Key Contrast	Corrected *p*	Interpretation
Side (/214)	GPT-5.4	184/214 86.0%	194/214 90.7%	206/214 96.3%	142/214 66.4%	B vs. D	1.37 × 10^−11^	Strong wrong-side bias
Side (/214)	Gemini	183/214 85.5%	209/214 97.7%	203/214 94.9%	122/214 57.0%	B vs. D	2.97 × 10^−19^	Even stronger wrong-side bias
Side (/214)	GPT-5.4	184/214 86.0%	-	206/214 96.3%	-	A vs. C	3.82 × 10^−6^	Context improved laterality
Side (/214)	Gemini	183/214 85.5%	-	203/214 94.9%	-	A vs. C	2.51 × 10^−4^	Context improved laterality
Displacement (/81)	GPT-5.4	48/81 59.3%	54/81 66.7%	60/81 74.1%	48/81 59.3%	A vs. C	0.033	Context improved displacement
Displacement (/81)	Gemini	34/81 42.0%	37/81 45.7%	33/81 40.7%	33/81 40.7%	A vs. C	1.00	No context effect

**Table 5:** How the prespecified statistical checks support the main interpretation.

Question	Main Result	Statistical Support	Why It Matters
Wrong-side bias	Laterality fell 90.7% to 66.4% in GPT and 97.7% to 57.0% in Gemini.	McNemar; both Holm *p* < 0.001.	Wrong context can sharply mislead both models.
Helpful context	Laterality improved from run A to run C in both models.	GPT Holm *p* = 3.82 × 10^−6^; Gemini Holm *p* = 2.51 × 10^−4^.	Correct context can improve broad tasks.
Displacement context effect	Only GPT improved for intracapsular displacement.	GPT Holm *p* = 0.033; Gemini Holm *p* = 1.00.	Context effects were task- and model-specific.
Detection difference	Gemini detected more fractures than GPT in runs E and F.	Holm *p* = 0.033 and 0.005.	Detection strength differed by model.
Complementarity	Union sensitivity was high, but detection agreement was limited.	Kappa 0.316 in run E; 0.093 in run F.	The models often failed on different cases.
Confidence warning	Confidence stayed high even when the answer was wrong.	Pattern medians: GPT 93 vs. 91; Gemini 95 vs. 95.	Displayed certainty was not a reliable safety cue.

## Data Availability

The data presented in this study are not publicly available due to patient privacy, ethical restrictions, and institutional data-sharing policies. The aggregated results supporting the findings are provided within the article and Appendix A. Additional aggregated or de-identified summary data may be made available by the corresponding author upon reasonable request and subject to institutional approval.

## Appendix A. Supplemental Methodological Notes

The analysis framework intentionally prioritized conceptually prespecified questions over exhaustive pairwise benchmarking. The primary questions were whether context changed output, whether wrong context could mislead, whether the models displayed complementary errors, and whether self-reported confidence behaved in a potentially misleading way. Union analyses were descriptive and hypothesis-generating; they were included to illustrate overlap and complementarity, not to estimate the performance of a deployable combined system.

All statistical analyses were performed in R 4.5.3. Wilson 95% confidence intervals were used for descriptive proportions because several denominators were modest in size and Wald intervals would have been less reliable. McNemar tests were used for paired within-case comparisons because the same radiographs were evaluated repeatedly under different prompting conditions. Holm correction was applied to the prespecified comparison family to reduce the risk of false-positive inference while avoiding the excessive conservatism of Bonferroni adjustment.

Cohen’s kappa was chosen to quantify inter-model agreement beyond raw percentage agreement, and weighted kappa was used for AO/OTA because it is an ordered multilevel variable. Confidence calibration was explored non-parametrically with Mann–Whitney tests because confidence scores were bounded and not assumed to be normally distributed.

**Table A1:** Full model performance matrix with Wilson 95% confidence intervals.

Run	Model	Detection	Side	Pattern	Displacement	AO/OTA/Subtroch.
A	GPT-5.4	-	86.0% (80.7–90.0)	79.4% (73.5–84.3)	59.3% (48.4–69.3)	27.0%/30.3%
A	Gemini	-	85.5% (80.2–89.6)	77.6% (71.5–82.6)	42.0% (31.8–52.8)	34.0%/51.5%
B	GPT-5.4	-	90.7% (86.0–93.9)	79.4% (73.5–84.3)	66.7% (55.9–76.0)	26.0%/24.2%
B	Gemini	-	97.7% (94.6–99.0)	79.0% (73.0–83.9)	45.7% (35.3–56.5)	35.0%/42.4%
C	GPT-5.4	-	96.3% (92.8–98.1)	77.6% (71.5–82.6)	74.1% (63.6–82.4)	23.0%/21.2%
C	Gemini	-	94.9% (91.0–97.1)	75.2% (69.0–80.5)	40.7% (30.7–51.6)	31.0%/30.3%
D	GPT-5.4	-	66.4% (59.8–72.3)	78.0% (72.0–83.1)	59.3% (48.4–69.3)	26.0%/27.3%
D	Gemini	-	57.0% (50.3–63.5)	73.8% (67.6–79.3)	40.7% (30.7–51.6)	32.0%/39.4%
E	GPT-5.4	87.9% (82.8–91.6)	79.0% (73.0–83.9)	71.0% (64.6–76.7)	38.3% (28.4–49.2)	23.0%/21.2%
E	Gemini	94.4% (90.5–96.8)	80.4% (74.5–85.1)	73.4% (67.1–78.8)	33.3% (24.0–44.1)	40.0%/45.5%
F	GPT-5.4	88.8% (83.9–92.3)	86.4% (81.2–90.4)	72.9% (66.6–78.4)	35.8% (26.2–46.7)	23.0%/15.2%
F	Gemini	97.2% (94.0–98.7)	93.5% (89.3–96.1)	74.8% (68.5–80.1)	38.3% (28.4–49.2)	37.0%/36.4%

**Table A2:** Prespecified paired McNemar comparisons (asterisks denote Holm-adjusted *p* < 0.05).

Comparison	b	c	Raw *p*	Holm-Adjusted *p*
GPT side B vs. D	52	0	1.522 × 10^−12^	1.37 × 10^−11^ *
GPT side A vs. C	0	22	4.768 × 10^−7^	3.82 × 10^−6^ *
GPT displacement A vs. C	3	15	0.00754	0.0331 *
GPT detection E vs. F	1	3	0.625	1.00
Gemini side B vs. D	87	0	2.966 × 10^−20^	2.97 × 10^−19^ *
Gemini side A vs. C	2	22	3.588 × 10^−5^	2.51 × 10^−4^ *
Gemini displacement A vs. C	17	16	1.00	1.00
Gemini detection E vs. F	2	8	0.109	0.328
Detection difference, run E (GPT vs. Gemini)	5	19	0.00661	0.0331 *
Detection difference, run F (GPT vs. Gemini)	4	22	0.000856	0.00514 *

**Table A3:** Selected inter-model agreement and confidence-calibration results.

Analysis	Result	*p* Value	Comment
Detection kappa, run E	0.316	0.0407	Limited agreement despite improved combined detection.
Detection kappa, run F	0.093	0.594	Very low agreement, supporting complementary misses.
GPT overall pattern confidence	Median 93 vs. 91 (correct vs. incorrect)	4.24 × 10^−21^	Some association, but confidence remains high when wrong
GPT overall side confidence	Median 93 vs. 88	1.14 × 10^−46^	Confidence higher when correct, but still high when wrong
Gemini overall pattern confidence	Median 95 vs. 95	1.76 × 10^−11^	Statistical difference without practical separation
Gemini overall side confidence	Median 95 vs. 95	0.523	No useful calibration for laterality
**Confidence warning**	**High confidence frequently persisted even when outputs were incorrect**	**-**	**Model self-confidence could not be treated as a reliable safety cue.**

**Table A4:** Cross-model overlap counts for fracture detection and detection with correct laterality in runs E and F. Counts are shown as both correct, GPT-only correct, Gemini-only correct, and neither correct. In runs E and F, laterality requires both fracture detection and correct side assignment.

Run	Outcome	Both Correct	GPT Only	Gemini Only	Neither Correct
E	Detection	183	5	19	7
E	Detection + correct laterality	149	20	23	22
F	Detection	186	4	22	2
F	Detection + correct laterality	178	7	22	7

## Appendix B. Verbatim Prompts and API Parameters

All prompts and API configurations used in this study are reproduced below verbatim. The system prompt was identical across all models and runs. Two task prompt templates were used: one for Runs A–D (fracture assumed present) and one for Runs E–F (fracture detection mode). Run-specific clinical context was prepended as noted.

**System prompt (all models, all runs):**

You are assisting in a research study. This is NOT for clinical care.

**Task prompt template—Runs A–D (fracture assumed present):**

Task: Using the provided AP pelvis/hip radiograph, determine the fracture SIDE first, then classify the hip fracture pattern. If the fracture is extracapsular, also specify whether it is intertrochanteric or subtrochanteric. Suggest a BROAD treatment category.

Return ONLY valid JSON with exactly these keys: {“fracture_side”: “left|right|uncertain”, “fracture_pattern”: “intracapsular|extracapsular|uncertain”, “intracapsular_displacement”: “displaced|nondisplaced|uncertain|not_applicable”, “extracapsular_location”: “intertrochanteric|subtrochanteric|uncertain|not_applicable”, “ao_ota_classification”: “31-A1|31-A2|31-A3|uncertain|not_applicable”, “treatment_category”: “fixation|arthroplasty|uncertain”, “cementation_note”: “cemented_stem_recommended|not_applicable|uncertain”, “confidence_0_100”: <integer 0-100>, “key_radiographic_signs”: [“<max 3 short phrases>”]}

Rules: 1. If findings are insufficient to decide, choose “uncertain” (do not guess). 2. Do not ask questions. Do not provide detailed operative planning. 3. Output MUST be raw JSON only (no markdown, no extra text). 4. If fracture_pattern is “extracapsular”, set “intracapsular_displacement” to “not_applicable”. 5. If fracture_pattern is “intracapsular”, set “extracapsular_location” to “not_applicable” AND set “ao_ota_classification” to “not_applicable”. 6. If fracture_pattern is “uncertain”, set “intracapsular_displacement” AND “extracapsular_location” AND “ao_ota_classification” to “uncertain”. 7. If fracture_pattern is “extracapsular”: (7.1) Set “extracapsular_location” to “intertrochanteric”/“subtrochanteric” (or “uncertain”). (7.2) If “intertrochanteric”, set “ao_ota_classification” to 31-A1/31-A2/31-A3 (or “uncertain”). (7.3) If “subtrochanteric”, set “ao_ota_classification” to “not_applicable”. (7.4) If “extracapsular_location” is “uncertain”, set “ao_ota_classification” to “uncertain”. 8. If treatment_category is NOT “arthroplasty”, set “cementation_note” to “not_applicable”.

**Task prompt template—Runs E–F (fracture detection mode):**

Task: You are provided with an AP pelvis/hip radiograph. There may or may not be a fracture. Carefully analyze the image and answer the following: 1. Is there a fracture visible? 2. If yes—which side (left/right)? 3. If yes—is it in the pelvis (intracapsular) or the femur/trochanteric region (extracapsular)? 4. If extracapsular—is it intertrochanteric or subtrochanteric? 5. If extracapsular intertrochanteric—provide AO/OTA classification (31-A1, 31-A2, or 31-A3). 6. Suggest a broad treatment category.

Return ONLY valid JSON with exactly these keys: {“fracture_present”: “yes|no|uncertain”, “fracture_side”: “left|right|uncertain|not_applicable”, “fracture_pattern”: “intracapsular|extracapsular|uncertain|not_applicable”, “intracapsular_displacement”: “displaced|nondisplaced|uncertain|not_applicable”, “extracapsular_location”: “intertrochanteric|subtrochanteric|uncertain|not_applicable”, “ao_ota_classification”: “31-A1|31-A2|31-A3|uncertain|not_applicable”, “treatment_category”: “fixation|arthroplasty|conservative|uncertain”, “cementation_note”: “cemented_stem_recommended|not_applicable|uncertain”, “confidence_0_100”: <integer 0-100>, “key_radiographic_signs”: [“<max 3 short phrases>”]}

Rules: 1. If no fracture is visible, set “fracture_present” to “no” and all other fields to “not_applicable”. 2. If uncertain whether a fracture exists, set “fracture_present” to “uncertain”. 3. If fracture_present is “yes”, apply the same rules as below for classification. 4. If fracture_pattern is “extracapsular”, set “intracapsular_displacement” to “not_applicable”. 5. If fracture_pattern is “intracapsular”, set “extracapsular_location” and “ao_ota_classification” to “not_applicable”. 6. If fracture_pattern is “extracapsular” and location is “intertrochanteric”, set AO/OTA to 31-A1/A2/A3 or “uncertain”. 7. If fracture_pattern is “extracapsular” and location is “subtrochanteric”, set “ao_ota_classification” to “not_applicable”. 8. If treatment_category is NOT “arthroplasty”, set “cementation_note” to “not_applicable”. 9. Output MUST be raw JSON only (no markdown, no extra text).

**Run-specific context prefixes:**

Run A: Image only (no clinical context prepended). Run B: “Clinical context: The patient presents with [correct side] hip pain.” Run C: “Clinical context: Painful side: [side] hip; Age: [age] years; Gender: [gender]; Mechanism: [mechanism]; Pre-injury ambulation: [ambulation]; Weight bearing before surgery: [weight_bearing]; Known osteoporosis: [osteoporosis].” Run D: “Clinical context: The patient presents with [wrong side] hip pain.” (deliberately reversed laterality). Run E: Image only (no clinical context prepended; detection mode). Run F: “Clinical context: The patient presents with [correct side] hip pain.” (detection mode).

**API parameters:**

GPT-5.4 (model identifier: gpt-5.4, resolved to snapshot gpt-5.4-2026-03-05): temperature = 0, max_completion_tokens = 500, image detail = high. Gemini 3.1 Pro (model identifier: gemini-3.1-pro-preview): temperature = 1 (vendor default; no zero-temperature option available), max_output_tokens = 16,384. Both models were accessed through their respective vendor APIs (OpenAI API and Google Generative AI API) in April 2026. Images were transmitted as base64-encoded PNG at native resolution.
